# Identifying Drug Candidates for COVID-19 with Large-Scale Drug Screening

**DOI:** 10.3390/ijms24054397

**Published:** 2023-02-23

**Authors:** Yifei Wu, Scott D. Pegan, David Crich, Lei Lou, Lauren Nicole Mullininx, Edward B. Starling, Carson Booth, Andrew Edward Chishom, Kuan Y. Chang, Zhong-Ru Xie

**Affiliations:** 1 School of Electrical and Computer Engineering, College of Engineering, University of Georgia, Athens, GA 30602, USA; 2 Division of Biomedical Sciences, School of Medicine, University of California Riverside, Riverside, CA 92521, USA; 3 Department of Pharmaceutical and Biomedical Sciences, College of Pharmacy, University of Georgia, Athens, GA 30602, USA; 4 Franklin College of Arts and Sciences, University of Georgia, Athens, GA 30602, USA; 5 Department of Computer Science and Engineering, National Taiwan Ocean University, Keelung 202, Taiwan

**Keywords:** COVID-19, virtual screening, PL protease, MD simulations, drug discovery

## Abstract

Papain-like protease (PL^pro^) is critical to COVID-19 infection. Therefore, it is a significant target protein for drug development. We virtually screened a 26,193 compound library against the PL^pro^ of SARS-CoV-2 and identified several drug candidates with convincing binding affinities. The three best compounds all had better estimated binding energy than those of the drug candidates proposed in previous studies. By analyzing the docking results for the drug candidates identified in this and previous studies, we demonstrate that the critical interactions between the compounds and PL^pro^ proposed by the computational approaches are consistent with those proposed by the biological experiments. In addition, the predicted binding energies of the compounds in the dataset showed a similar trend as their IC_50_ values. The predicted ADME and drug-likeness properties also suggested that these identified compounds can be used for COVID-19 treatment.

## 1. Introduction

With more than 756 million cases and 6.8 million deaths worldwide as of February 2023, the SARS-CoV-2 (COVID-19) pandemic is one of the most significant pandemics in recent history [1]. In the United States, deaths due to this virus have surpassed the 1918 H1N1 pandemic, the most lethal flu pandemic in the last century [2,3,4]. To curb these numbers, vaccines against COVID-19 have been in development since the sequencing of the virus in early 2020 [5,6]. As of the writing of this article, many vaccines have been approved worldwide and more are still in development: Pfizer-BioNTech and Moderna in the United States, Oxford-AstraZeneca in the United Kingdom, Covaxin in India, etc. [7]. Despite this success, cases continue to surge in the US, Japan, Germany, and many other high-income nations [8]. Although they have not experienced as many infections, low-income nations are disproportionally underserved by current vaccines, resulting in extended restrictions on their populations [7]. To ease the negative impact of these issues, continued drug development for COVID-19 is very important.

Drugs developed for COVID-19 vary in size and primarily target one of three viral proteins: RNA-dependent RNA polymerase (RdRp), 3C-like protease (3CL^pro^ or M^pro^), and papain-like protease (PL^pro^) [9,10]. Remdesivir, the first COVID-19 drug approved by the FDA, is an adenine analog nucleoside inhibitor that disables RdRp by incorporating itself into the new sequence and blocking transcription [11,12]. Molnupiravir (EIDD-2801) and Favipiravir are further drugs of this type in development [9,10,11]. The next most prominent drug in development is PF-07321332, developed by Pfizer, which binds to the active site of 3CL^pro^ using a series of hydrogen bonds, with Glu166, Gln189, and His164 of 3CL^pro^ being the most important residues anchoring and stabilizing the ligand throughout the binding process [10,13]. Its mechanism is similar to Boceprevir, which inhibits COVID-19 3CL^pro^ and has been approved by the FDA to treat HCV [10,14]. On a separate note, many antibodies, such as REGEN-COV and Sotrovimab, approved by the FDA to treat mild COVID-19 have different targets from the aforementioned proteins [9].

PL^pro^ is a well-known cysteine protease. PL^pro^ not only cleaves non-structural proteins 1–3 (nsp1–3) from polyprotein 1ab, a major protein involved in early COVID-19 infection [10,15,16], but also aids in immune evasion by cleaving ubiquitin (Ub) and interferon (IFN)-stimulated gene 15 (ISG15), which are significant parts of the NF-κB pathway. Near the catalytic site, the last four residues of Ub bind to leucine, arginine, and two glycines, respectively, which are known as P1–P4. Most of the above substrates are anchored by P1–P4 before cleavage by the catalytic site [10]. Along with this, PL^pro^ plays a role in attaching the replication/transcription complex (RTC) to the modified endoplasmic reticulum (ER) membranes [15].

This wide range of functions makes PL^pro^ an attractive drug target. Most drugs against PL^pro^ prevent Ub and ISG15 from reaching the catalytic site. GRL-0617, the most prominent drug candidate against PL^pro^, binds to P3 and P4 using H-bonds and hydrophobic interactions between Tyr265, Tyr 269, Pro248, and Pro249. GRL-0667, a more potent inhibitor (IC_50_ = 0.32 μM), utilizes a similar mechanism, with its smaller size and conformation allowing for greater interaction with the binding site [10]. Some derivatives of GRL-0617’s functional groups are more potent than GRL-0617, such as XR8-89 (IC_50_ = 0.113 μM) and XR8-83 (IC_50_ = 0.21 μM) [16]. Acting as a Zn^2+^ ejector and disrupting the interaction with Cys111, Ebselen also inhibits PL^pro^ with an IC_50_ of 0.67 ± 0.09 μM [10,17]. Regarding virtual screening studies, cyanobacterial metabolites, such as Cryptophycin 1 (−7.7 ± 0.09 kcal/mol), and compounds from natural sources, such as matcha green tea and Nigella sativa, have good binding affinity when docked to PL^pro^, indicating likely inhibitory activity as well [18,19,20,21].

To push drug development further, we screened a 26,193 compound library for potential candidates against PL^pro^ using molecular docking and molecular mechanics with generalized Born and surface area (MM-GBSA) calculations. While the potential candidates were compared to GRL-0617 and our previous best compound, the top three candidates were selected to undergo further molecular dynamics (MD) simulations. Overall, this study identified three novel drug candidates (F3077-0136, F2883-0639, and F0514-5148) against PL^pro^ that have lower binding affinities than the control compounds, potentially surpassing them in inhibitory power.

## 2. Results

### 2.1. Docking Analysis of Top Three Compounds against SARS-CoV-2 PL^pro^

To develop effective drugs against SARS-CoV-2 PL^pro^, we docked 26,193 compounds on PL^pro^ (PDB ID: 7LBR) by performing ligand–protein docking. Based on the docking results, the binding energies were calculated using Prime MM-GBSA in Maestro. As a result, the top three compounds with the best binding energies were found to be F3077-0136 (−91.92 kcal/mol), F2883-0639 (−90.96 kcal/mol), and F0514-5148 (−89.66 kcal/mol) (Table 1). Moreover, a reported PL^pro^ inhibitor (GRL-0617) [22,23], the best drug candidate kaempferol 3-O-sophoroside 7-O-glucoside from our previous study [21], and the existing ligand XR8-89 in 7LBR were used as controls. From Table 1, it can be seen that these three compounds show better binding energies than the control compounds, indicating that these top three compounds may be drug candidates against SARS-CoV-2 PL^pro^.

**Figure 1:**
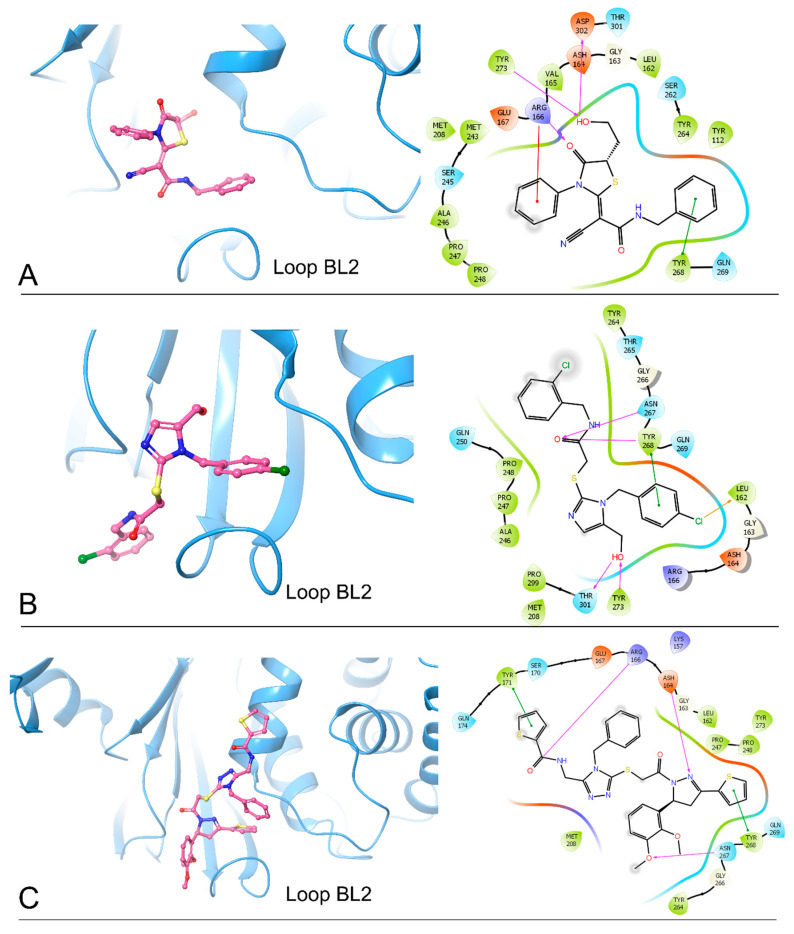
The docking poses and 2D ligand–protein interaction diagrams for 7LBR and the top three ligands: (**A**) F3077-0136; (**B**) F2883-0639; (**C**) F0514-5148. The pink arrow indicates the hydrogen bond; the green line represents pi–pi stacking; the red line represents pi–cation interaction; the yellow line represents the halogen bond.

From 2D ligand–protein interaction diagrams (Figure 1), we found that the top three compounds could all interact with Tyr268. F3077-0136 and F0514-5148 interact with Tyr268 by each forming one pi–pi stacking. F2883-0639 interacts with Tyr268 by forming one hydrogen bond and one pi–pi stacking. Furthermore, both F2883-0639 and F0514-5148 interact with Asn267 by each forming one hydrogen bond. Notably, Asn267 and Tyr268 are the residues on blocking loop 2 (BL2), which plays an essential role in inhibitor binding [24]. This potentially explains why these top three compounds can tightly bind to PL^pro^.

Additionally, F3077-0136 interacts with Arg166 by forming a hydrogen bond and a pi–cation interaction. F0514-5148 also interacts with Arg166 by forming a hydrogen bond. Both F3077-0136 and F2883-0639 interact with Tyr273 by each forming one hydrogen bond. All the interactions between the top three compounds and the residues in the binding pocket are summarized in Table 2. We also calculated the strength of the hydrogen bonds between the top three compounds and the residues (Table S1), and the data indicated that the hydrogen bonds between the top three compounds and the binding residues were strong.

### 2.2. Physicochemical Properties Prediction

To explore the physicochemical properties of the top three compounds, we predicted the absorption, distribution, metabolism, excretion (ADME), and the drug-likeness properties by using Qikprop in Maestro. The results are shown in Table 3. First, the molecular weights and the QPlogS of the top three compounds were in the recommended ranges of 130.0 to 725.0 and −6.5 to 0.5, respectively. F3077-0136 and F2883-0639 showed better solubility than F0514-5148 because the QPlogS value of F0514-5148 was close to −6.5. In addition, Lipinski’s rule of five (RO5) and Jorgensen’s rule of three (RO3) were used to evaluate the drug-likeness. Lipinski’s rule of five (RO5) requires molecular weight < 500, an octanol–water partition coefficient (LogP) < 5, the number of hydrogen bond donors ≤ 5, and the number of hydrogen bond acceptors ≤ 10 [25]. Jorgensen’s rule of three (RO3) requires aqueous solubility (LogS) > −5.7, the apparent Caco-2 cell permeability > 22 nm/s, and the number of primary metabolites < 7 [26]. All three compounds passed the RO5 and RO3 tests, while both F3077-0136 and F2883-0639 showed no RO5 and RO3 violations and F0514-5148 had two violations for each test. These results demonstrate that these three compounds are potential drug candidates. Therefore, F3077-0136, F2883-0639, and F0514-5148 were studied further.

### 2.3. Molecular Dynamics (MD) Simulation Analysis

**Figure 2:**
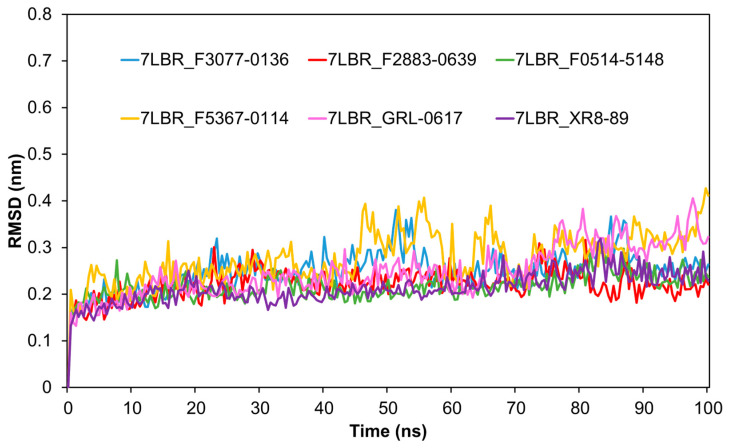
The comparison of the proteins’ RMSD values. The blue, red, green, yellow, pink, and purple lines represent the RMSD values for 7LBR after binding to F3077-0136, F2883-0639, F0514-5148, F5367-0114 (negative control), GRL-0617 (drug candidate from a previous study), and the cocrystal structure 7LBR_XR8-89, respectively.

**Figure 3:**
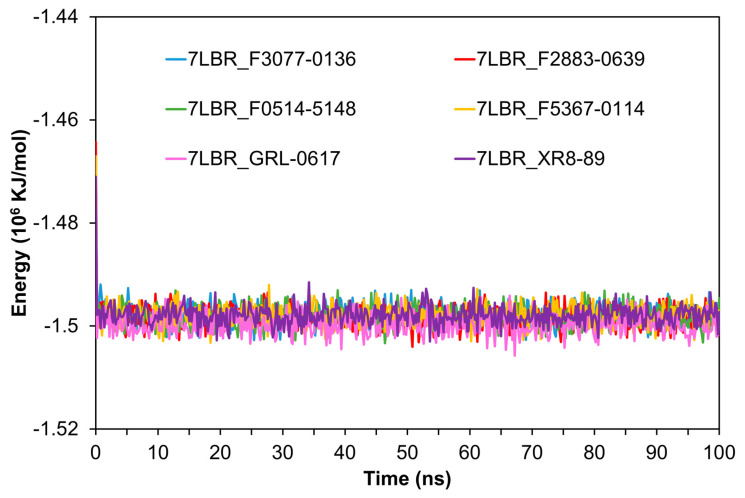
The comparison of the total energies of the protein–ligand complexes. The blue, red, green, yellow, pink, and purple lines represent the energies of the complexes 7LBR_F3077-0136, 7LBR_F2883-0639, 7LBR_F0514-5148, 7LBR_F5367-0114, and 7LBR_GRL-0617 and the cocrystal structure 7LBR_XR8-89, respectively.

To further analyze the stability of PL^pro^ bound to the top three compounds, we conducted MD simulations to calculate the root-mean-square deviation (RMSD) for the proteins (Figure 2), the total energies (Figure 3), the H-bond (Figure S5), the radius of gyration (rGyr) (Figure S6), the solvent-accessible surface area (SASA) (Figure S7), and the root-mean-square fluctuation (RMSF) (Figure S8). RMSD can be used to evaluate the stability of a protein structure. As shown in Figure 2, the RMSD of 7LBR bound to F2883-0639 and F0514-5148 stabilized around 0.25 nm from 4 to 100 ns, similar to the RMSD for the cocrystal structure 7LBR-XR8-89. This suggested that the protein structures of 7LBR bound to F2883-0639 and F0514-5148 were stable. The RMSD of 7LBR bound to F3077-0136 ranged from 0.20 to 0.38 nm from 4 to 90 ns and then stabilized around 0.25 nm after 90 ns. Moreover, the complex 7LBR-F5367-0114, which showed worse binding energy (−29.81 kcal/mol), was applied as a negative control. The RMSD of 7LBR-F5367-0114 stabilized around 0.25 nm from 5 to 45 ns and then ranges from 0.3 to 0.4 nm until 100 nm. As the positive control, the RMSD of 7LBR bound to GRL-0617 ranged from 0.20 to 0.30 nm from 4 to 75 ns and then rose higher than 0.30 nm (Figure 2). Both the negative and positive controls showed higher RMSD values than the top three complexes. Therefore, we concluded that the proteins bound to the top three compounds were more stable than the proteins bound to F5367-0114 and GRL-0617. The total energies of the top three complexes and the control complexes are shown in Figure 3. The energies of the 7LBR–ligand complexes stabilized at around −1.49 × 10^6^ KJ/mol, which also indicated the good stabilities of these systems. In addition, as depicted in Figure S5, 7LBR bound to F3077-0136 exhibited a higher number of hydrogen bonds than the other proposed complexes and was even better than the cocrystal structure 7LBR-XR8-89 from 0 to 60 ns, indicating the higher stability of 7LBR-F3077-0136. In Figure S6, the values for rGyr can be seen to stabilize around 2.35 to 2.43 nm, except for the negative control 7LBR-F5367-0114, indicating that the three proposed complexes have similar flexibilities compared to the positive control and the cocrystal structure. Furthermore, the SASA analysis plot (Figure S7) shows that the SASA values of the top three complexes were in the range of 159 nm^2^ –172 nm^2^, similar to the positive control and the cocrystal structure. This suggests that the top three complexes have similar solvent accessibilities as those of the positive control and the cocrystal structure. Notably, as shown in Figure S8, the top three complexes exhibited similar RMSF distributions compared to the positive control and the cocrystal structure, indicating their steady binding stabilities.

## 3. Discussion

COVID-19 has led to the largest economic, educational, and societal disruptions of any pandemic seen throughout history, with an estimated USD 3.3 trillion deficit within the United States and USD 202.6 billion in lost revenue throughout American healthcare systems [27]. Hence, drug development for the treatment of COVID-19 has accelerated drastically. PL^pro^ is one of the critical therapeutic targets for COVID-19 treatment. Numerous studies have proposed PL^pro^ inhibitors, such as GRL-0617 and its derivative, XR8-89 [16,28]. GRL-0617 is not an ideal antiviral agent due to its insufficient potency [16]; however, it can be used as a control for virtual screening. The cocrystal structure (PDB ID: 7LBR) of XR8-89 with SARS-CoV PL^pro^ was applied for screening in this study. The existing ligand XR8-89 was used as another control. As one of the derivatives of GRL-0617, XR8-89 showed better binding energy than GRL-0617 (Table 1), which was consistent with their IC_50_ data (the IC_50_ of GRL-0617 is 1.61 µM and the IC_50_ of XR8-89 is 0.113 µM) [16]. The binding energy of XR8-89 was close to those of the top three candidates, which indicates that the top three candidates should have similar or better inhibitory potency against PL^pro^, as a previous study acknowledged that binding energy is a significant indicator of drug potency [29]. Furthermore, in addition to XR8-89, we tested the other compounds listed in the paper by Shen et al. [16] and compared the estimated binding energies with IC_50_ values. As can be seen in Figure S1, our predicted binding energies showed a similar trend as the IC_50_ values, suggesting the accuracy of the docking results.

In addition, there were six more candidates with better binding energies than the control group (Table S2). The binding energies of those candidates were around −88 kcal/mol. By comparing the protein–ligand interactions, we found that five of them interacted with Asn267, Tyr268, and Gln269 (Table S3). These three residues belong to the BL2 loop (Gly266-Gly271) which is an essential structure in PL^pro^ for inhibitor binding [24]. Therefore, F3222-1354, F1827-0078, F3166-0259, F3222-3821, and F1614-0151 may exert inhibitory effects on PL^pro^. Among these five compounds, the Qikprop descriptors for F1827-0078, F3166-0259, and F1614-0151 fell within the recommended range, suggesting that these three compounds may be used for COVID-19 treatment (Table S4). To further validate our results, we are also testing these candidates using biochemical methods in our collaborative lab, and the outcomes will be published in the future once our collaborators complete the experiments.

## 4. Materials and Methods

### 4.1. Ligand Preparation

The structures of the 26,193 tested compounds were provided by the College of Pharmacy, University of Georgia. Before preparation, we analyzed this database using DataWarrior [30]. More than 90% of the compounds had molecular weights (MWs) below 450 (MW ≤ 450) (Figure S2), and around 96% of the compounds showed good hydrophilicity (cLogP ≤ 5) (Figure S3). After calculating the drug-likeness values in DataWarrior, around 70% of the compounds fell within the recommended range (drug-likeness value > 0) (Figure S4). All the tested compounds were prepared using Ligprep in Maestro 12.4 (Schrödinger). The process employed by Ligprep involves adding hydrogen molecules, computing the correct partial charges, and generating possible conformations. The force field used was OPLS3e by default [31].

### 4.2. Protein Preparation

The protein structure of SARS-CoV-2 PL^pro^ (PDB ID: 7LBR [16]) from RCSB’s Protein Data Bank (https://www.rcsb.org/, accessed on 20 April 2022) [32] was prepared using Schrodinger Maestro. The protein preparation included three steps [33]. The first step was preprocessing, which included assigning bond orders, adding hydrogen, creating zero-order bonds to metals, creating disulfide, and generating het states using Epik [34]. The second step was optimization, which involved optimizing the hydrogen bond using PROPKA [35]. The third step was minimization, which was performed by using the OPLS3e force field [31].

### 4.3. Ligand–Protein Docking

To estimate the interactions between target proteins and the tested compounds, we conducted ligand–protein docking by using the Ligand Docking panel in Maestro. Before running docking jobs, a receptor grid box was generated based on the existing ligand XR8-89 (Ligand ID: XT7) in the structure 7LBR [16]. The size of the receptor grid box was set as the default (20 Å). Ligand–protein docking was performed as flexible docking in extra-precision (XP) mode.

### 4.4. MM-GBSA Calculation

To predict the binding energies of the tested compounds bound to PL^pro^, we performed Prime molecular mechanics with generalized Born and surface area (MM-GBSA) calculations in Maestro. The pose-viewer files generated after dockings were uploaded into the Prime MM-GBSA panel to calculate binding energy. The force field used was OPLS3e [31].

### 4.5. ADME and Drug-Likeness Properties Prediction

After the calculation of binding energies, we applied the Qikprop module in Maestro to predict absorption, distribution, metabolism, excretion (ADME), and drug-likeness properties for further screening [36]. For Qikprop, the top three compounds were prepared using Ligprep. Finally, the descriptors, such as the rule of five (RO5) and rule of three (RO3), were applied to analyze the candidates.

### 4.6. Molecular Dynamics Simulations

To further investigate the dynamic interactions between PL^pro^ and the top three compounds, we conducted molecular dynamics (MD) simulations using GROMACS version 2030.4 and CHARMM36 force field [37,38]. The starting coordinates for the protein–ligand complexes were obtained from our ligand–protein docking studies. Then, we used CHARMM-GUI to build the MD simulation solution box, which was a cubic box with a length of 112 Å, and filled it with water molecules [39]. Next, the minimized structures were equilibrated using a constant number of particles, volume, and temperature (NVT) ensemble and a number of particles, pressure, and temperature (NPT) ensemble. The target equilibration temperature was 303.15 K. Finally, MD simulations were performed for 100 ns and the conformations were calculated and recorded every 2 ps. During the simulations, the simulation solution box was isotropic and the periodic boundary condition was applied. After the MD simulations, we calculated the root-mean-square deviation (RMSD) for the protein and the energies of the system along the simulation trajectories.

## 5. Conclusions

This study demonstrated that F3077-0136, F2883-0639, and F0514-5148 were the best three drug candidates. In particular, F3077-0136 and F2883-0639 showed better drug-likeness properties. In this study, we applied several computational approaches to a 26,193 compound library provided by the College of Pharmacy, University of Georgia. All compounds were available for the ensuing assays. Before the biological assays, we docked all 26,193 compounds on PL^pro^, predicted the binding affinities to identify the best inhibitors, predicted the drug-likeness, and performed MD simulations to validate the predicted binding. Overall, this study not only provides clues for bench research but may also speed up the process of COVID-19 drug development.

## Figures and Tables

**Table 1:** The estimated binding energies of the top three compounds and the control compounds.

Compound	Structure	Binding Energy (kcal/mol)
F3077-0136	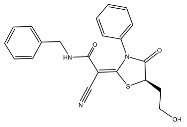	−91.92
F2883-0639	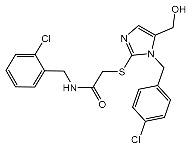	−90.96
F0514-5148	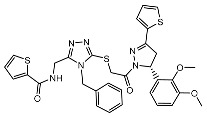	−89.66
GRL-0617	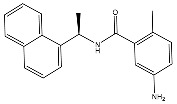	−60.75 [21]
Kaempferol 3-O-sophoroside 7-O-glucoside	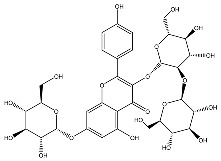	−87.97 [21]
XR8-89	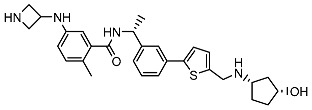	−87.41

**Table 2:** The interactions between the top three compounds and the residues of SARS-CoV-2 PL^pro^ and their distances (H-bond: hydrogen bond).

Residue	F3077-0136	F2883-0639	F0514-5148
Leu162		Halogen bond (3.33 Å)	
Asp164			H-bond (1.87 Å)
Arg166	H-bond (2.29 Å, 2.05 Å)		H-bond (2.31 Å)
	Pi–cation (4.58 Å)		
Tyr171			Pi–pi stacking (4.82 Å)
Asn267		H-bond (2.03 Å)	H-bond (2.02 Å)
Tyr268	Pi–pi stacking (4.64 Å)	H-bond (2.67 Å)	Pi–pi stacking (5.45 Å)
		Pi–pi stacking (4.78 Å)	
Tyr273	H-bond (1.85 Å)	H-bond (1.75 Å)	
Thr301		H-bond (2.71 Å)	
Asp302	H-bond (2.03 Å)		

**Table 3:** Selected Qikprop descriptors for the top three compounds and the control compounds.

Compound	mol_MW ^1^	QPlogS ^2^	RO5 ^3^	RO3 ^4^
F3077-0136	393.459	−5.404	0	0
F2883-0639	436.355	−5.440	0	0
F0514-5148	658.807	−6.367	2	2
GRL-0617	304.391	−4.952	0	0
F5367-0114	372.433	−4.230	0	0

^1^ mol_MW represents the molecular weights of the molecules. The recommended range is 130.0–725.0. ^2^ QPlogS is the predicted aqueous solubility. The recommended range is −6.5~0.5. ^3^ RO5: number of violations of Lipinski’s rule of five. The maximum is four. ^4^ RO3: number of violations of Jorgensen’s rule of three. The maximum is three.

## Data Availability

Publicly available data was generated in this study. This data can be found at: https://sourceforge.net/projects/msdock/.

## Supplementary Materials

The following supporting information can be downloaded at: https://www.mdpi.com/article/10.3390/ijms24054397/s1.
