# Supplementary material for: Identifying Drug Candidates for COVID-19 with Large-Scale Drug Screening
Source: Int J Mol Sci. 2023 Feb 23;24(5):4397. doi: 10.3390/ijms24054397 (PMC10002104; doi:10.3390/ijms24054397)
Supplement: Supplementary file 1 [file ijms-24-04397-s001.zip › ijms-2171909-supplementary.pdf]

Supplementary Material

Table S1. H-bond strength of the top three compounds and the residues (kcal/mol)

Residue	F3077-0136	F2883-0639	F0514-5148
Asp164			H-bond (-7.74)
Arg166	H-bond (-32.92)		H-bond (-12.53)
Asn267		H-bond (-9.23)	H-bond (-3.67)
Tyr268		H-bond (-9.58)	
Tyr273	H-bond (-23.59)	H-bond (-10.86)	
Thr301		H-bond (-9.68)	
Asp302	H-bond (-31.52)		

Table S2. The other six candidates with better binding energies than the control group

Compound	Binding Energy (kcal/mol)
F3166-0258	-88.56
F3222-1354	-88.52
F1827-0078	-88.40
F3166-0259	-88.34
F3222-3821	-88.17
F1614-0151	-88.07

Table S3. The interactions between the six compounds and the residues on BL2 loop

Compound	Asn267	Tyr268	Gln269
F3166-0258	-	-	-
F3222-1354	H-bond	H-bond<br>pi-pi stacking	H-bond
F1827-0078	-	pi-pi stacking<br>pi-pi stacking	-
F3166-0259	-	pi-pi stacking<br>pi-pi stacking	H-bond
F3222-3821	H-bond	H-bond	-
F1614-0151	-	H-bond<br>pi-pi stacking<br>pi-pi stacking	-

“-” represents no interaction

Table S4. Selected Qikprop descriptors of the six compounds

Compound	mol_MW[1]	QPlogS[2]	RO5[3]	RO3[4]
F3166-0258	578.026	-8.023	2	1
F3222-1354	418.512	-6.764	0	1
F1827-0078	424.517	-5.231	0	0
F3166-0259	549.025	-3.535	1	0
F3222-3821	551.634	-8.871	2	1
F1614-0151	432.499	-5.697	0	0

1 mol_MW represents molecular weight of the molecule. The recommended range is 130.0–725.0.
2 QPlogS is the predicted aqueous solubility. The recommended range is −6.5~0.5.
3 RO5: number of violations of Lipinski's rule of five. The recommended range: maximum is 4.
4 RO3: number of violations of Jorgensen's rule of three. The recommended range: maximum is 3.

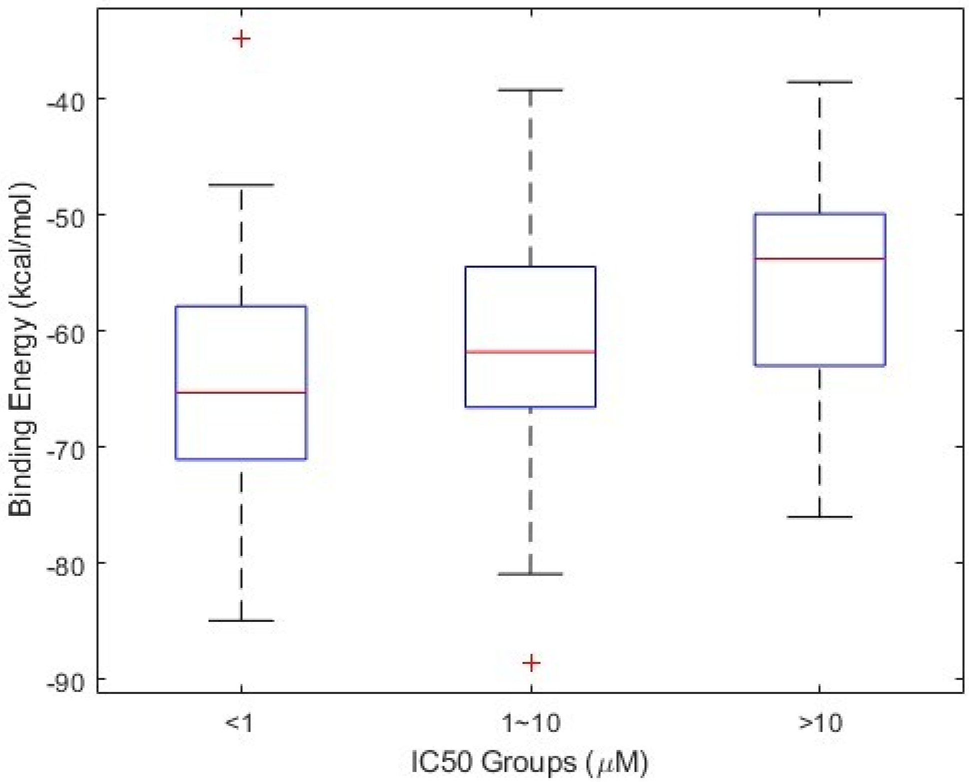

Figure S1. Box plots of binding energies of the compounds published in the paper [16] categorized by IC_{50} groups.

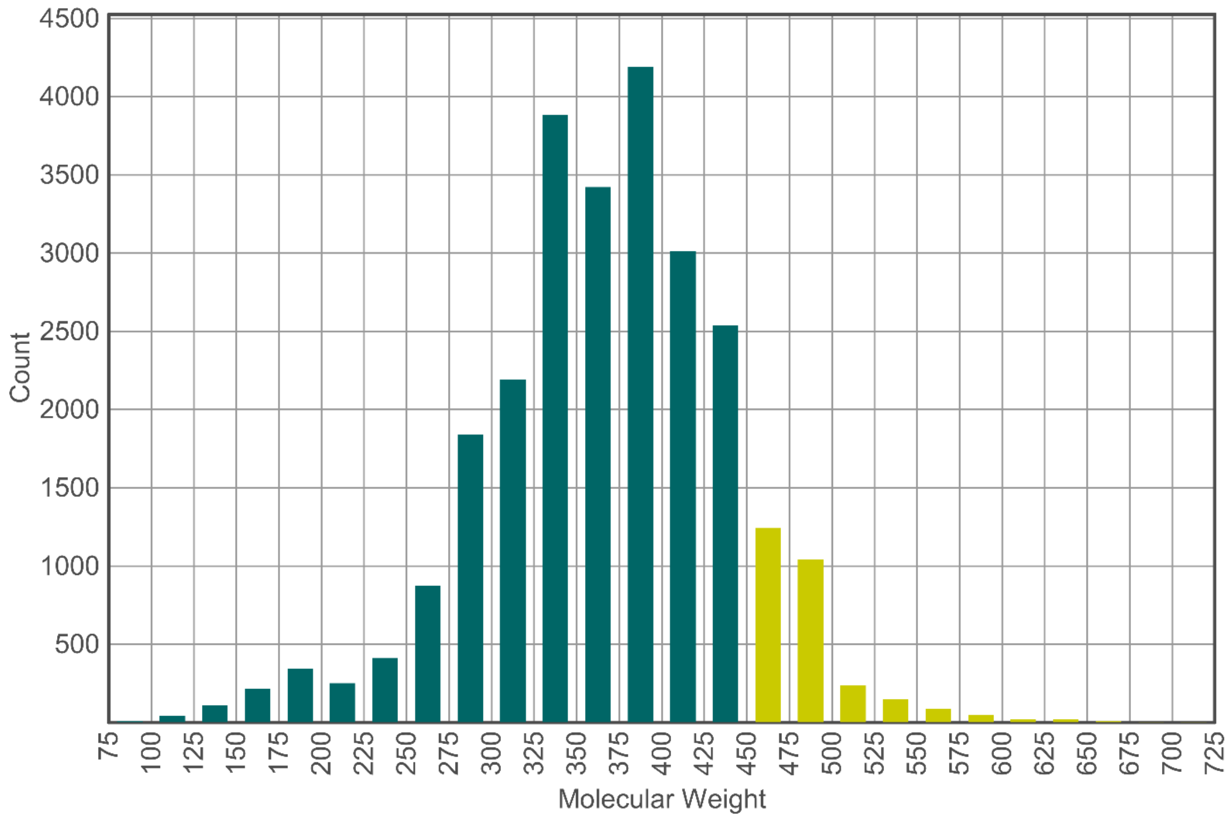

Figure S2 Distribution of molecular weight in the tested compounds. The green part represents that the data falls within the recommended range.

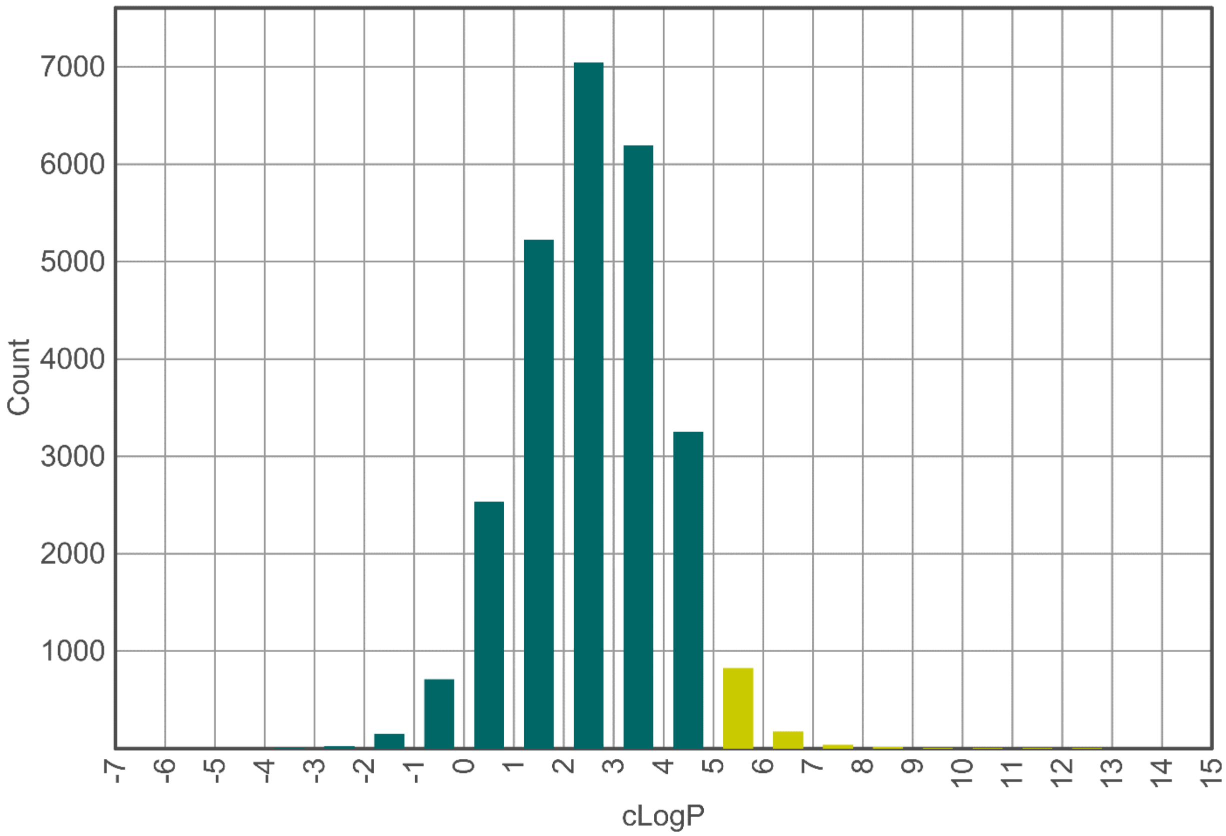

Figure S3 Distribution of calculated LogP in the tested compounds. The green part represents that the data falls within the recommended range.

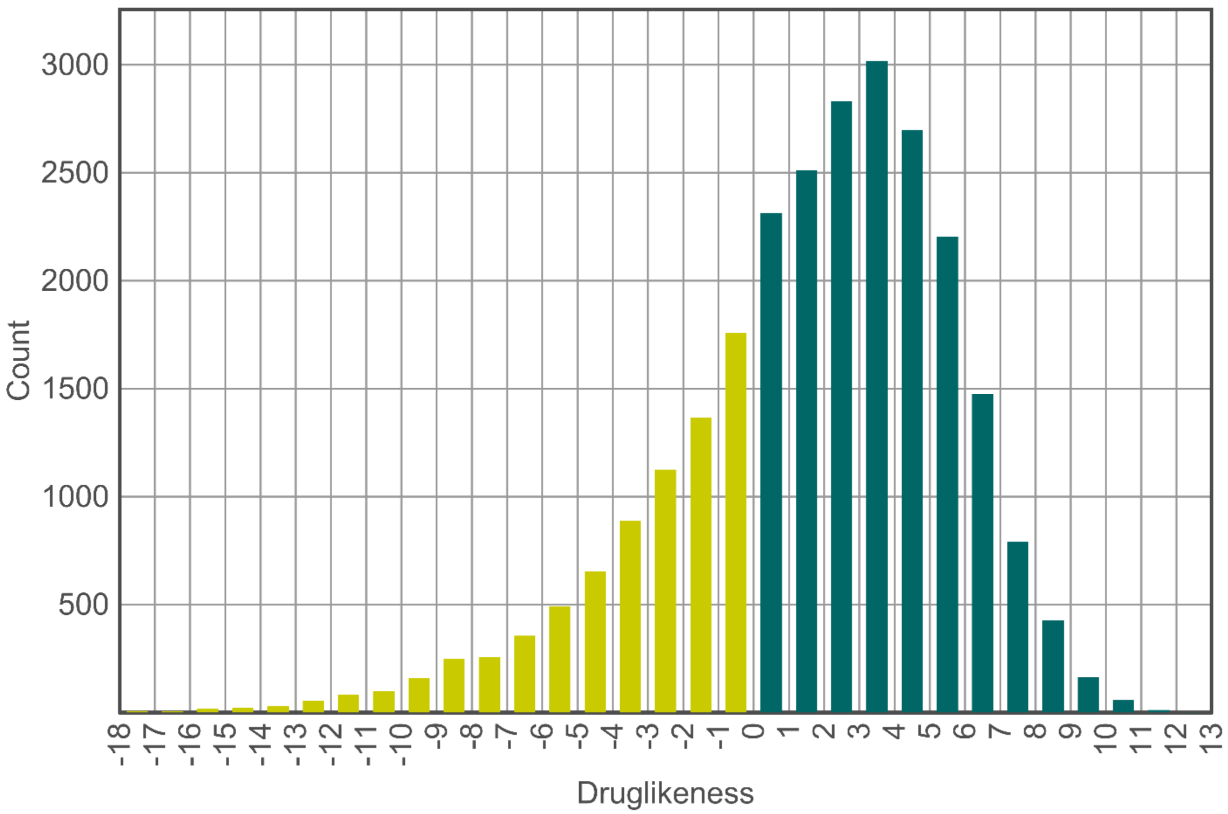

Figure S4 Distribution of druglikeness values in the tested compounds. The green part represents that the data falls within the recommended range.

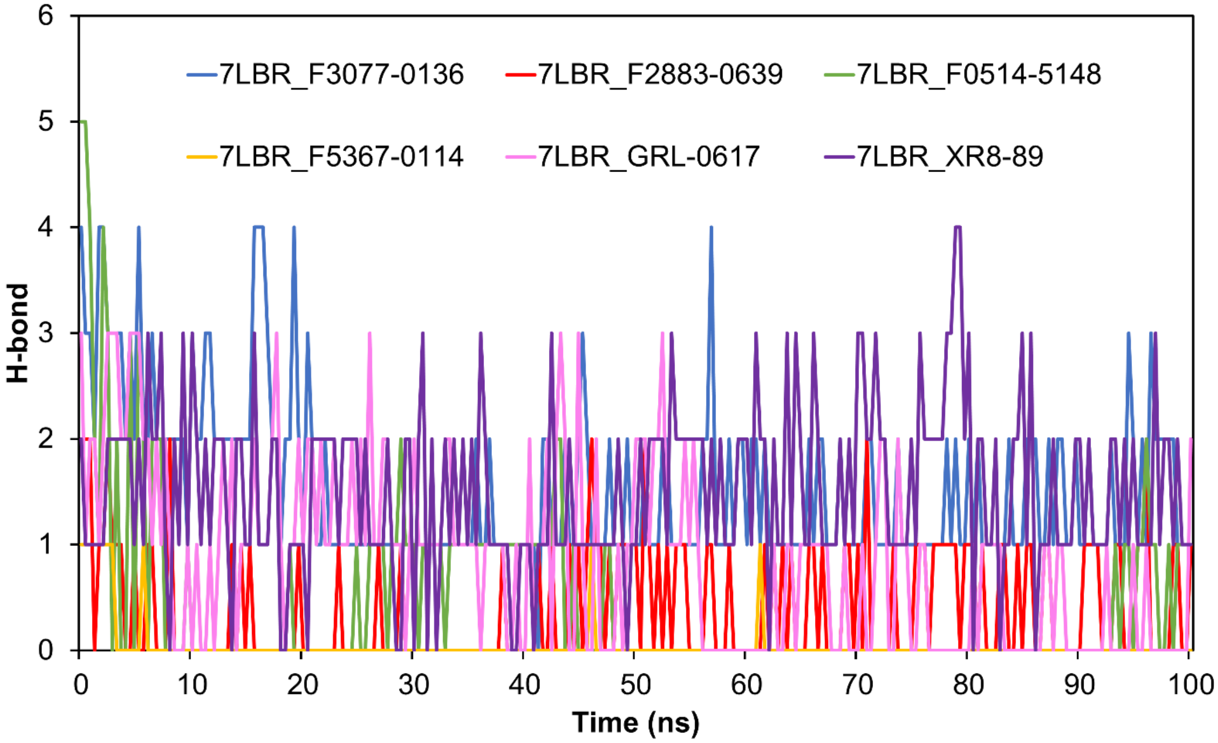

Figure S5 The comparison of H-bond of protein-ligand complexes. The blue, red, green, yellow, pink, and purple lines represent the energies of complexes: 7LBR_F3077-0136, 7LBR_F2883-0639, 7LBR_F0514-5148, 7LBR_F5367-0114, 7LBR_GRL-0617, and the cocrystal structure 7LBR_XR8-89, respectively.

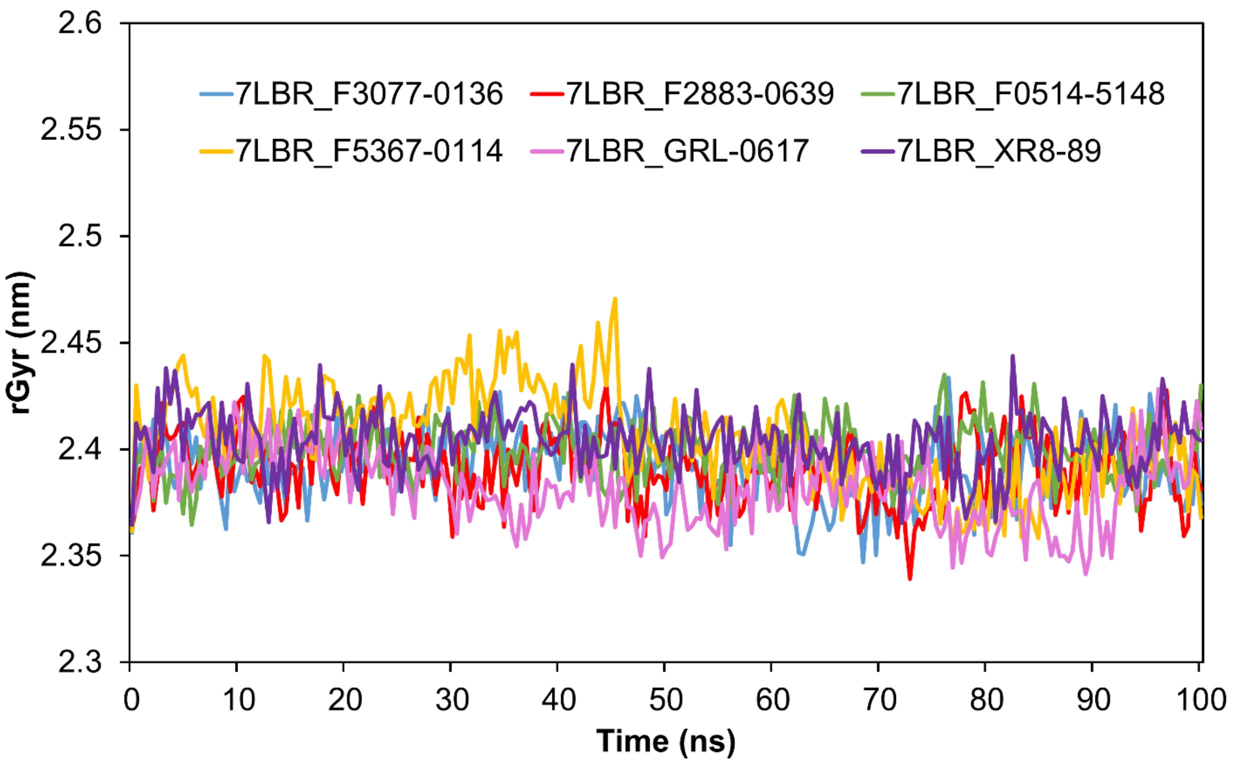

Figure S6 The comparison of the radius of gyration (rGyr) of protein-ligand complexes. The blue, red, green, yellow, pink, and purple lines represent the energies of complexes: 7LBR_F3077-0136, 7LBR_F2883-0639, 7LBR_F0514-5148, 7LBR_F5367-0114, 7LBR_GRL-0617, and the cocrystal structure 7LBR_XR8-89, respectively.

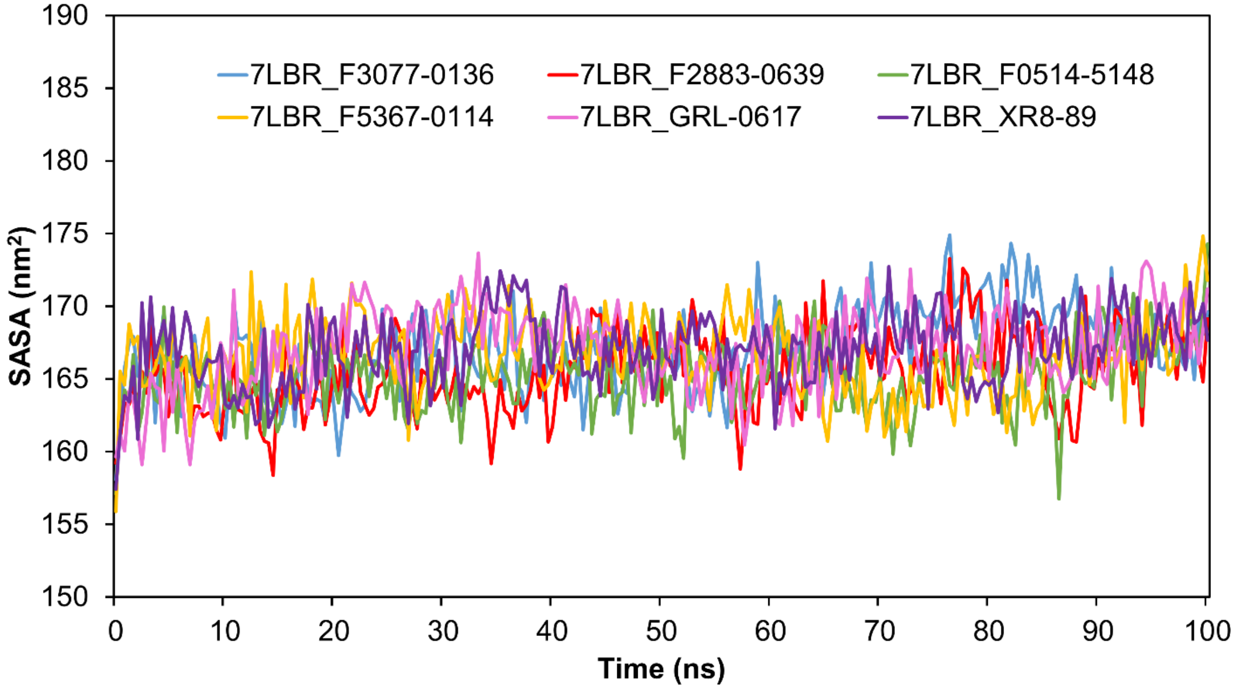

Figure S7 The comparison of the solvent accessible surface area (SASA) of protein-ligand complexes. The blue, red, green, yellow, pink, and purple lines represent the energies of complexes: 7LBR_F3077-0136, 7LBR_F2883-0639, 7LBR_F0514-5148, 7LBR_F5367-0114, 7LBR_GRL-0617, and the cocrystal structure 7LBR_XR8-89, respectively.

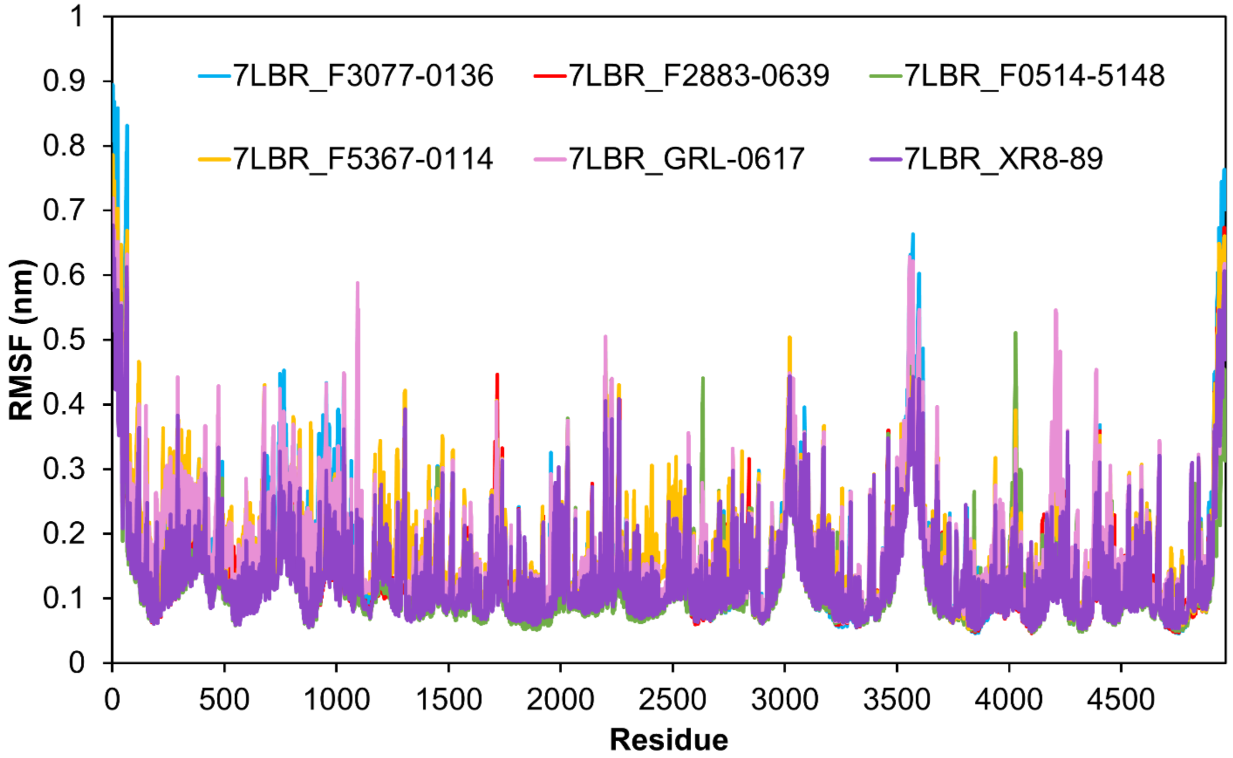

Figure S8 The comparison of the root-mean-square fluctuation (RMSF) of protein-ligand complexes. The blue, red, green, yellow, pink, and purple lines represent the energies of complexes: 7LBR_F3077-0136, 7LBR_F2883-0639, 7LBR_F0514-5148, 7LBR_F5367-0114, 7LBR_GRL-0617, and the cocrystal structure 7LBR_XR8-89, respectively.
